# Sailing to Gondwana: multiple waves of spinosaurid dispersal to the southern continents

**DOI:** 10.1098/rsos.260994

**Published:** 2026-10-28

**Authors:** Zak E. Lewis, Charlie R. Scherer, Tom T. P. van der Linden, Paul J. Byrne, Bethany Burke, Colin Boisvert, Ezekiel V. O'Callaghan, Kenneth C. Rayburn, Robert Mayer, Chance Guest, Harry T. Jones, Tristan Moran, Steven J. R. Allain, Cassius Morrison

**Affiliations:** ^1^ School of Earth Sciences, University of Bristol, Bristol, UK; ^2^ Department of Earth Sciences, University College London, London, UK; ^3^ Freie Universität Berlin, Berlin, Germany; ^4^ Het Nationaal Oertijdmuseum, Boxtel, The Netherlands; ^5^ Department of Anatomy, New York Institute of Technology College of Osteopathic Medicine, Old Westbury, USA; ^6^ The Heritage Museum of the Texas Hill Country, USA; ^7^ Oklahoma State University Centre for Health Sciences, Tulsa, USA; ^8^ Department of Biology, Northern Arizona University, Flagstaff, USA; ^9^ Department of Earth and Environmental Sciences, Columbia University, New York, USA; ^10^ Independent Researcher, UK; ^11^ School of Biological and Marine Sciences, University of Plymouth, Plymouth, UK; ^12^ School of Geography, Earth and Environmental Sciences, University of Birmingham, Birmingham, UK; ^13^ Sparsholt College, Winchester, UK; ^14^ Writtle School of Agriculture, Animal and Environmental Sciences, Anglia Ruskin University, Chelmsford, Essex, UK

**Keywords:** biogeography, dinosaur, Cretaceous, Spinosauridae, climate, dispersal, Spinosaurinae, Baryonychinae, Megalosauroidea

## Abstract

Spinosaurid theropods exhibited a relatively widespread distribution during the Cretaceous Period; however, biogeographical analyses have been limited, largely owing to their fragmentary nature and uncertainties in spinosaurid phylogeny. We conducted a biogeographical analysis of Megalosauroidea, with a focus on Spinosauridae, using the R package ‘BioGeoBEARS’ on multiple phylogenetic trees to test their probable dispersal patterns. The best-fit models suggest that spinosaurid evolutionary history was influenced by geodispersal followed by narrow and widespread vicariance, as well as regional sympatry between co-occurring taxa. Within Spinosauridae, we find at least two distinct dispersals from Europe into Africa and South America, as well as at least one dispersal from Europe into Asia. Based on existing climate model data, spinosaurids generally appear to occur in high mean annual temperature semi-aquatic environments (approx. 26.1–33.1°C) with relatively minimal seasonal variation (approx. ±8.46°C), which may have influenced their dispersal capability, as these preferences appearmore constrained compared with those of other medium-to-large theropods. These results imply a more limited biogeographical distribution for Spinosauridae compared with other clades, which may be owing to climatic dispersal barriers and environmental factors.

## 1. Introduction

Megalosauroidea is a clade of medium-to-large-sized carnivorous dinosaurs (greater than or equal to 100 kg; [1–5]), including the first named dinosaur, *Megalosaurus bucklandii* [6,7], as well as the charismatic African giant *Spinosaurus aegyptiacus* [8]. Spinosauridae, a family confidently known from the Cretaceous Period [9,10], is conventionally subdivided into two morphologically distinct clades: Baryonychinae, which contains the genera *Baryonyx*, *Suchomimus*, *Ceratosuchops* and *Riparovenator*, are defined by their serrated, slightly recurved teeth and smaller size, whereas Spinosaurinae, which includes *Irritator*, *Oxalaia* and *Spinosaurus*, are characterized by their unserrated, conical teeth, larger size and nostrils positioned further up the skull [11–14]. The ecology of spinosaurids is one of the most contested aspects of theropod research, especially for the genus *Spinosaurus*, with some studies hypothesizing a subaqueous lifestyle [5,15–19] and others hypothesizing a more ‘heron-like’, terrestrial lifestyle [10,20–24]. Spinosaurids appear to be found almost exclusively in fluvial, deltaic, and lagoonal sedimentary deposits (table 1).

**Table 1.** Summary of depositional environments for the main spinosaurid genera investigated.

Species	Age (Ma)	Continent	Formation	Sedimentology	Depositional environment
*Baryonyx walkeri*	130–125	Europe	Weald Clay	Dark grey shale, light grey mottled silty clay, topped with laterally variable sandstone beds [25]	Fluvial mudplain [26]
*Suchomimus tenerensis*	125–112	Africa	Elhraz	Medium-grained fluvial sandstone [27]	Fluvial [11]
*Ceratosuchops inferodios*	130–125	Europe	Wessex	Sandstones and varicoloured mudstones with interspersed plant debris beds [28]	Alluvial, fluvial and possibly lacustrine [28]
*Riparovenator milnerae*	130–125	Europe	Wessex	Sandstones and varicoloured mudstones with interspersed plant debris beds [28]	Alluvial, fluvial and possibly lacustrine [28]
*Camarillasaurus cirugedae*	130–125	Europe	Camarillas	Light brown clay and limestone rocks with fossil wood remains [29]	Fluvial [29]
*Iberospinus natarioi*	129.4–125	Europe	Papo Seco	Silty mud-mud deposition and green silty clays, with lignitic horizons and gypsum; interbedded with medium to coarse, commonly ribbon-shaped, clastic sandstones [30]	Lagoonal to estuarine [30]
*Riojavenatrix lacustris*	125–113	Europe	Enciso Group	Mixed siliciclastic–carbonates [31]	Lacustrine [31]
*Vallibonavenatrix cani*	129.4–125	Europe	Arcillas de Morella	Red and green shale, with intercalations of sandstone lenticular bodies; abundant thin intercalations of sandy limestones, with ostracods and charophytes [32]	Estuarine-Delta complex [32]
*Ichthyovenator laosensis*	120–113	Asia	Grès supérieurs	Found within a layer of red sandstone [33]	Fluvial [34]
*Irritator challengeri*	113–110	South America	Romualdo [35,36]	Mudstone and concretionary limestone [37]	Lagoonal [37]
*Spinosaurus aegyptiacus*	100–94	Africa	Kem Kem Group	Variably fine sand, silt and clay. Millimetric-scale lamination sometimes present, suggestive of ‘dinoturbation’ or wet sediment deformation. Occasional bivalves distinct from freshwater unionids [38]	Palustrine [38]
*Spinosaurus aegyptiacus*	100–95	Africa	Bahariya	Conglomerates, sandstone, claystones and shale with some paleosols present [39]	Mangrove tidal flat; possible fluvial and/or floodplain settings [40]
*Spinosaurus mirabilis*	100–95	Africa	Farak	Siltstone and sandstone [23]	Riparian [23]
*Sigilmassasaurus brevicollis*	100–94	Africa	Kem Kem Group	Variably fine sand, silt and clay. Millimetric-scale lamination sometimes present, suggestive of ‘dinoturbation’ or wet sediment deformation. Occasional bivalves distinct from freshwater unionids [38]	Palustrine [38]
Gara Samani taxon	100.5–93.9	Africa	Gara Samani	Fine to coarse-grained, moderately to poorly sorted, quartz sands, interbedded with sandstone, clay, chenalized quartz pebbles and conglomerate [41]	Fluvial and aeolian [41]
*Oxalaia quilombensis*	100.5–93.9	South America	Alcântara	Sandstones, shales and minor conglomerates [42]	Tidal estuarine environment [43]
White rock spinosaur	126–125	Europe	Vectis	Laminated, cross-laminated or burrow-mottled sandstone with lenses of fusain-rich carbonaceous sandstone, organic-rich mudstones and poorly sorted conglomerate [44]	Fluvial to lagoonal [44]

However, spinosaurids are not ubiquitous across all continents, with a notable absence of these theropods in North America, Australia [45,46] and Antarctica [10]. Furthermore, North America preserves a low megalosauroid diversity, limited to only the megalosaurid *Torvosaurus* [47] and potentially the piatnitzkysaurid *Marshosaurus* [48,49]. Several competing hypotheses have been proposed regarding spinosaurid biogeography, focusing specifically on their origins and dispersal routes [28,50–52]. One hypothesis suggests a European origin followed by Early Cretaceous dispersal into Asia and South America and two separate dispersals into Africa, resulting in the presence of *Suchomimus* and *Spinosaurus* [28]. Alternatively, Sereno *et al.* [11] suggested that the widening of the Tethys Ocean separated an ancestral spinosaurid population. This separation was hypothesized to have induced the independent evolution of baryonychines in Laurasia and spinosaurines in Gondwana, with the former undergoing a single known dispersal into Africa, with the arrival of *Suchomimus* [11]. Here, we quantitatively examine the biogeographic evolution of Megalosauroidea, with an emphasis on spinosaurids, their potential dispersal routes and barriers to dispersion.

## 2. Material and methods

We extracted our dataset of megalosauroid occurrences from the Paleobiology Database (https://paleobiodb.org/, accessed September 2025), including stratigraphic provenance and geological age (supplementary material) and verified these within the literature. The majority of spinosaurid taxa are represented by incomplete remains, which complicates phylogenetic studies [10,12]. Consequently, we excluded *Ostafrikasaurus* [53] and other dubious taxa [54–59] from our main analysis due to a lack of phylogenetic data. The literature provided additional occurrences, with the final dataset consisting of 15 spinosaurids and 12 non-spinosaurid megalosauroids, ranging from the Toarcian (approx. 180 Ma) to the Cenomanian in age (approx. 95 Ma).

Owing to the unstable placement of various spinosaurids within phylogenetic analyses, a total of six alternative topologies were constructed in Mesquite (v.3.5.1; [60]) to account for this variation [31,44,51,61]. Polytomies were randomly resolved using the ‘multi2di’ function from the ‘*ape*’ R package (v5.8-1; [62]) to permit biogeographical analysis. To resolve the biogeographic history of Megalosauroidea, we used the R package ‘*BioGeoBEARS’* (v.1.1.3; [63]). All taxa were assigned to at least one of the five defined geographic regions (Europe, Asia, Africa, South America and North America) based on our occurrence data. To account for the uncertainty in the connectivity of the landmasses, we adopted two sets of dispersal matrices from Mannion *et al.* [64] that were applied to each tree, resulting in 12 biogeographic scenarios. The best-fitting model was recovered from each simulation according to the lowest sample-corrected Akaike Information Criterion (AIC), a model selection tool which simultaneously provides a measure of model fit whilst punishing model complexity [65,66]. Each of the three models within BioGeoBEARS considers the following biogeographic processes, each with and without a jump dispersal parameter (J): DEC/DEC+J models narrow and subset sympatry, with narrow vicariance; DIVALIKE/DIVALIKE+J models narrow sympatry only, but allows narrow and widespread vicariance events; BAYAREALIKE/BAYAREALIKE+J models narrow and widespread sympatry, but disallows vicariance. The ages and continental occurrences of all spinosaurids, including contentious and fragmentary specimens, were also sourced from a literature review (supplementary material) and plotted onto an inclusive taxonomic range chart in RStudio ([67], v4.5.1) using the ‘*deeptime’* package [68], as these specimens may also potentially hold important biogeographic implications [69].

Environmental data for all spinosaurid occurrence data were sourced from Chiarenza *et al.* [70], who used the HadCM3L Global Circulation Model to reconstruct palaeoclimatic variables (i.e. mean annual temperature, MAT; seasonal temperature range, STR; mean annual precipitation, MAP and seasonal precipitation range, SPR) within discrete stage-level bins for dinosaurian occurrences from the Jurassic and Cretaceous. Here, we extract a subset of climatic variables according to the occurrences of major theropod clades spanning the Berriasian–Cenomanian, where most unambiguous spinosaurid taxa occur, to demonstrate whether the family demonstrates a strong affinity for certain environmental conditions, which can offer key insight into their dispersal barriers.

## 3. Results

### 3.1. Biogeographic analyses

All analyses, independent of the differing spinosaurid topologies and dispersal parameter matrices tested, recovered robust support for the DIVALIKE (trees 1–3) and DIVALIKE + J (trees 4–6) models over all others (table 2). Continental-scale vicariance was the predominant driver of megalosauroid biogeographic evolution, the latter model highlighting the importance of the founder effect when *Riojavenatrix* is positioned as the earliest spinosaurine. DIVALIKE receives greater support than its +J variant (AIC_wt_ = 0.41 versus 0.31; table 2), and the ancestral spinosaurid is reconstructed as European, emerging from an ancestrally widespread megalosauroid population. A spinosaurine dispersal event from Europe to Asia is recovered as early as the Berriasian (approx. 143.1–137.05 Ma), with this lineage leading to *Ichthyovenator* (figures 1 and 2). At least two independent dispersals of spinosaurids occurred from Europe into Africa within or by the Barremian (approx. 125–121 Ma; figures 1 and 2), one within Baryonychinae and another in Spinosaurinae, followed by another two spinosaurine dispersals from Africa into South America by the Cenomanian (approx. 100–94 Ma),with the arrival of *Irritator* and *Oxalaia* (figure 2).

**Table 2.** Summary statistics from the BioGeoBEARS analysis using tree topology 1 and dispersal matrix 1 (see supplementary information for other analyses). LnL, log-likelihood.

		Ln likelihood ratio		AIC analysis		
Model	LnL	D-statistic	*p*-value	AIC	AIC_wt_	Ratio
**DIVALIKE**	–**55.03**	**-**	**-**	**114.5**	**0.41**	**-**
DIVALIKE+J	–54.03	2	0.16	115	0.31	0.780
DEC+J	–54.86	2.57	0.11	116.6	0.14	1.077
DEC	–56.14	-	-	116.7	0.13	-
BAYAREALIKE+J	–65.3	10.92	0.0010	137.5	4.1e-06	68.3
BAYAREALIKE	–70.76	-	-	146	6.0e-08	-

**Figure 1.**
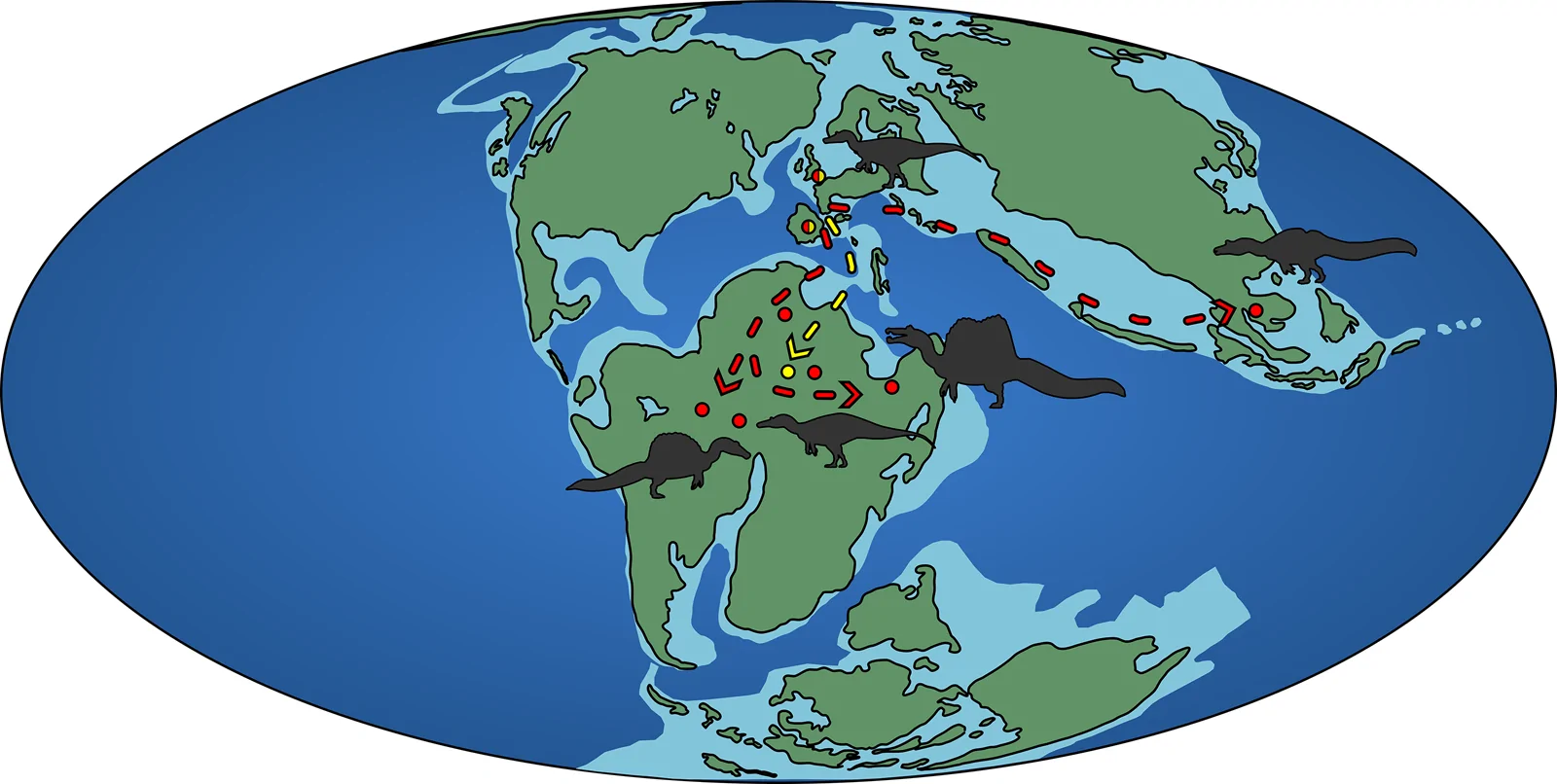
Probable pathways for the dispersal of Spinosauridae. Dispersal pathways relate to spinosaurines (red) and baryonychines (yellow). The dispersal routes were reconstructed using BioGeoBEARS and plotted on an Early Aptian (approx. 121.8 Ma) base map redrawn from Scotese [71]. Points indicate where spinosaurid taxa occur in the BioGeoBEARS dataset. Silhouettes vectorized from Connor Ashbridge (CC BY-SA 4.0), available from Wikimedia Commons images.

**Figure 2.**
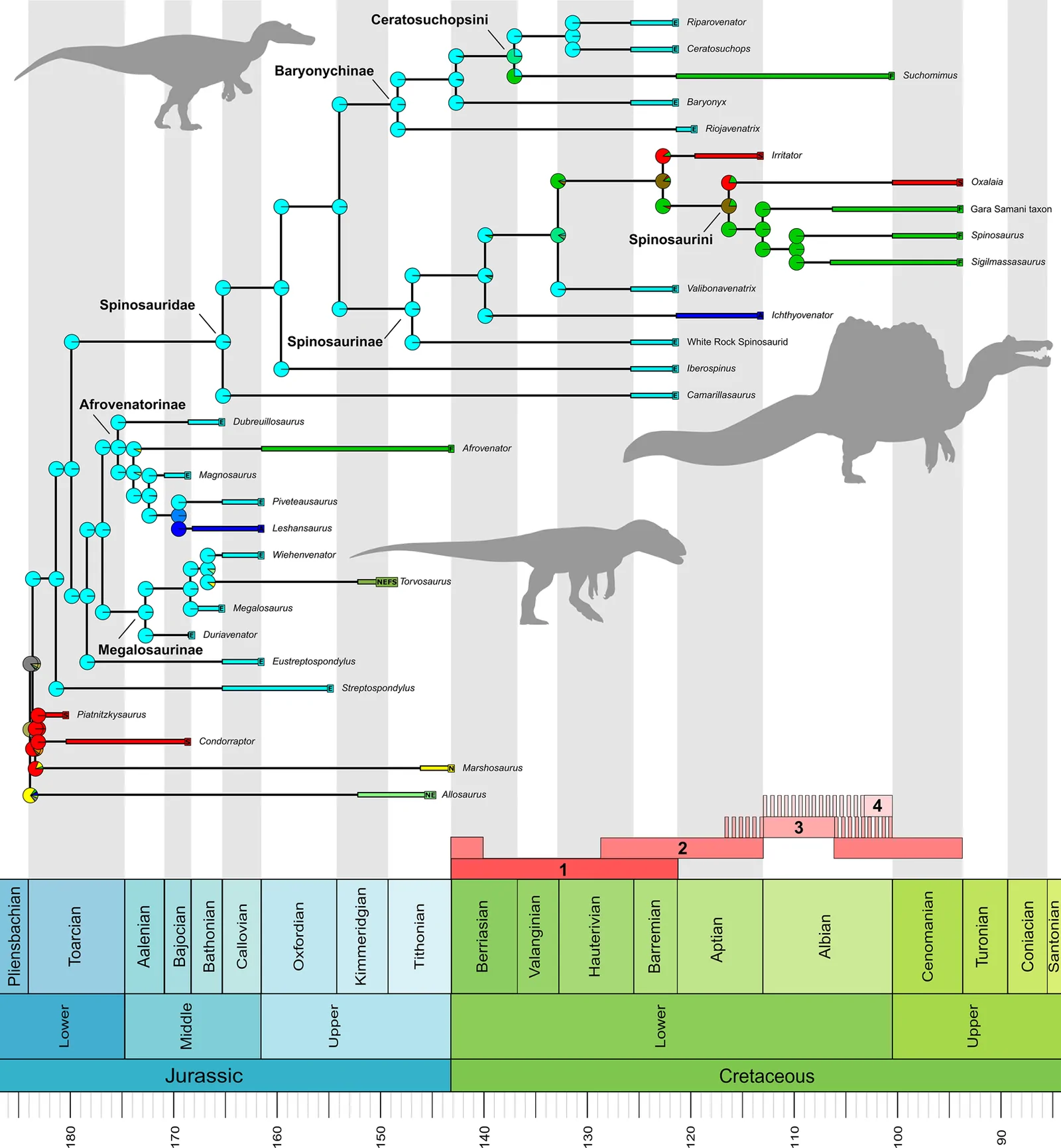
The palaeobiogeographic history of Megalosauroidea, showcasing the results of the DIVALIKE model, one of the two best-fitting models (see supplementary information for other analyses). Nodes relate to the most likely ancestral ranges of most recent common ancestors. The tips of each branch relate to each taxon's temporal range, while their colours and letters reflect the continent(s) from which each taxon inhabits. The numbered boxes represent the presence of certain dispersal routes through the Early Cretaceous, and broken boxes represent uncertainty in the timing of these routes. Key: (1) the Apulian route connecting Europe and Africa, (2) periods of marine regression of the Uralian seaway connecting Europe and Asia, (3) the Bering land bridge connecting Asia and North America, and (4) the separation of Africa and South America. E = Europe; A = Asia; N = North America; S = South America; F = Africa. The colours for the nodes and tips are light blue = Europe; dark blue = Asia; green = Africa; red = South America; yellow = North America; mixed = several continents. Silhouettes vectorized from Connor Ashbridge (*Baryonyx* and *Spinosaurus*; CC BY-SA 4.0) and Fred Wierum (*Torvosaurus*; CC BY-SA 4.0), both available from Wikimedia Commons images.

### 3.2. Spinosaurid climate ranges

Spinosaurids occurred in regions with high MAT (median = 28.8°C; interquartile range (IQR) = 26.1–33.1°C), appearing to be significantly warmer and more constrained compared with contemporary medium-to-large theropods (figure 3A), including carcharodontosaurians (median = 26.1°C; IQR = 18.3–31.0°C) and abelisaurids (median = 22.6°C; IQR = 16.6–31.1°C). This statistical pattern implies that, unlike spinosaurids, other contemporary large theropods were able to occupy cooler, higher-latitude regions [72,73]. Furthermore, spinosaurids appear to have an affinity for regions where seasonal temperature shifts are relatively minimal (median = 8.46°C; IQR = 5.26–12.1°C) and more constrained (figure 3B) compared with carcharodontosaurians (median = 11.1°C; IQR = 4.15–26.6°C) and abelisaurids (median = 13.3°C; IQR = 5.47–26.6°C). Conversely, we find no significant relationship between spinosaurid occurrences and MAP (median = 811 mm yr^–1^; IQR = 260–1051 mm yr^–1^) or SPR (median = 3.36 mm day^–1^; IQR = 1.41–6.24 mm day^–1^), which are broadly consistent across all sampled theropods (figure 3C,D, table 3; supplementary material, table S12). The recovered medians and ranges do not strictly reflect the inhabited conditions for every individual of a clade, but capture the broad-scale patterns of each group based on localized climate data of fossil localities.

**Figure 3.**
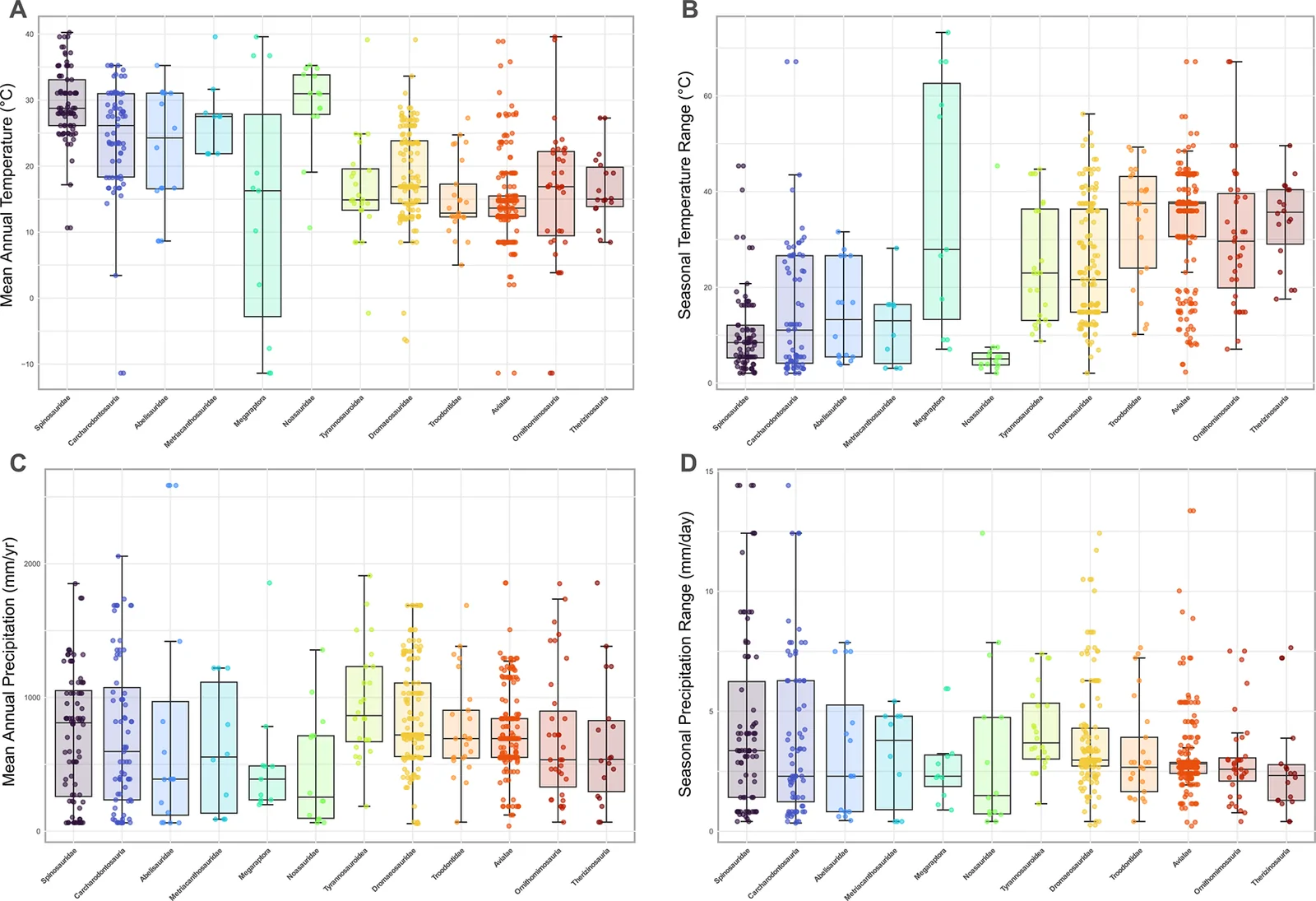
Climatic environments of Spinosauridae compared with contemporary Cretaceous theropods from the Berriasian–Cenomanian, based on data from [70]. (A) MAT in °C, (B) STR in °C, (C) MAP in mm yr^–1^ and (D) SPR in mm day^–1^.

**Table 3.** Summary statistics of different climatic ranges inhabited by theropods in the Berriasian–Cenomanian. MAT = mean annual temperature; IQR = interquartile range; STR = seasonal temperature range; MAP = mean annual precipitation; SPR = seasonal precipitation range. The data used come from Chiarenza *et al*. [70].

Clade	MAT (median)°C	MAT (IQR)°C	STR (median)°C	STR (IQR)°C	MAP (median) mm yr^–1^	MAP (IQR) mm yr^–1^	SPR (median) mm day^–1^	SPR (IQR) mm day^–1^
Spinosauridae	28.8	26.1–33.1	8.46	5.26–12.1	811	260–1051	3.36	1.41–6.24
Carcharodontosauria	26.1	18.3–31.0	11.1	4.15–26.6	596	234–1074	2.30	1.23–6.28
Abelisauridae	24.3	16.6–31.1	13.3	5.47–26.6	390	120–969	2.30	0.817–5.26
Metriacanthosauridae	27.5	21.9–27.9	13.0	4.09–16.4	555	135–1114	3.79	0.899–4.80
Megaraptora	16.3	–2.80–27.8	27.9	13.3–62.6	390	234–488	2.30	1.87–3.18
Noasauridae	31.0	27.8–33.8	5.05	3.78–6.29	255	96.9–714	1.49	0.728–4.75
Tyrannosauroidea	14.9	13.3–19.6	23.0	13.1–36.4	865	669–1231	3.68	3.01–5.34
Dromaeosauridae	16.9	14.3–23.8	21.6	14.8–36.3	720	558–1108	2.97	2.72–4.30
Troodontidae	12.9	12.3–17.3	37.5	24.0–43.2	692	547–905	2.67	1.65–3.92
Avialae	13.6	12.4–15.5	37.5	30.6–37.8	692	552–842	2.81	2.41–2.87
Ornithomimosauria	16.9	9.45–22.2	29.6	19.9–39.6	534	330–898	2.59	2.09–3.06
Therizinosauria	15.0	13.9–19.8	35.7	29.0–40.4	537	296–827	2.33	1.29–2.78

## 4. Discussion

### 4.1. Comparisons with previous hypotheses

Our biogeographic modelling illustrates that vicariance predominantly influenced megalosauroid evolutionary history, with at least one spinosaurid dispersal from Europe into Asia, and at least two dispersals from Europe into Africa and later from Africa to South America. Sereno *et al.* [11] initially hypothesized that spatial heterogeneity occurred between Baryonychinae in Laurasia and Spinosaurinae in Gondwana, with the occurrence of *Suchomimus* in Africa representing a single dispersal event from Laurasia. Yet, the discovery of Laurasian spinosaurine material, such as *Ichthyovenato*r from Laos [33] and possibly the ‘white rock spinosaurid’ from the Isle of Wight, UK [44], casts doubt on this hypothesis, suggesting spinosaurines were not exclusive to Gondwana. We find evidence of spinosaurines and baryonychines in Laurasia and Gondwana (figures 1, 2 and 4), with a possible Middle Jurassic European origin for Spinosauridae similar to previous studies [28,74,75]. Serrano-Martínez *et al.* [57] found a Middle Jurassic African origin for Spinosauridae with the description of MUPE HB-87 (a tooth from the Middle Jurassic Niger), however,its referral to Spinosauridae has since been disputed [57,76,77]; hence we excluded it from our BioGeoBEARS analyses (see Material and Methods for more details). The waves of spinosaurid dispersal towards Gondwana and eastern Laurasia, most likely from Europe, support the findings of Barker *et al.* [28] and provide evidence for at least periodic connectivity between these landmasses during the Early Cretaceous (figures 1 and 2). These patterns of connectivity have also been demonstrated by other Early Cretaceous dinosaurian clades, including coelurosaurian theropods [78,79] and eusauropods [64]. Yet, the apparent absence of spinosaurids from landmasses, such as North America, Antarctica and Australia, when other dinosaurian clades dispersed into those continents suggests there were unique and selective barriers for spinosaurid dispersal.

**Figure 4.**
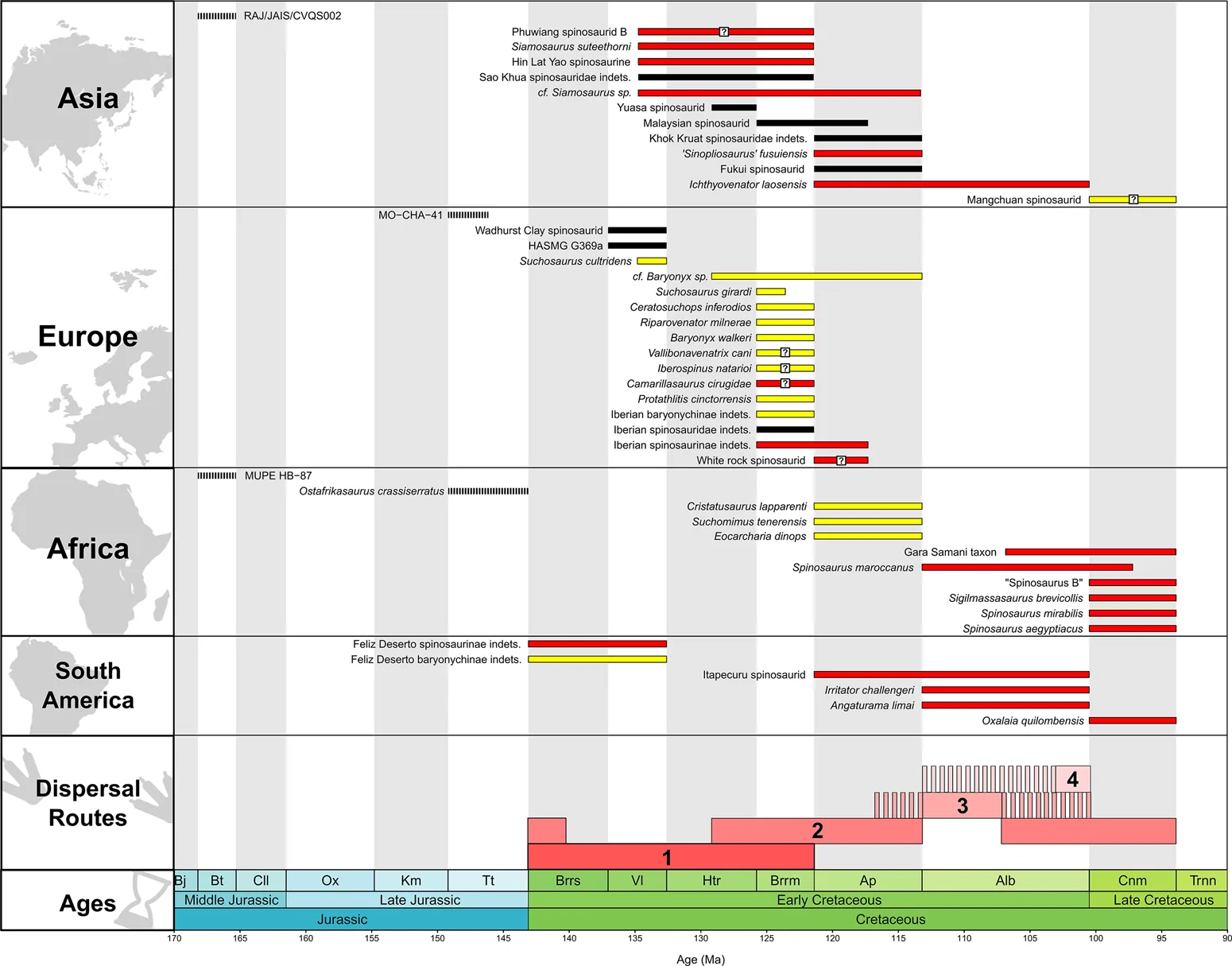
An inclusive taxonomic range chart of Spinosauridae, including fragmentary and early dubious specimens within their geographic context. The numbered boxes represent the presence of certain dispersal routes through the Early Cretaceous, and broken boxes represent uncertainty in the timing of these routes. Key: (1) the Apulian route connecting Europe and Africa, (2) periods of marine regression of the Uralian seaway connecting Europe and Asia, (3) the Bering landbridge connecting Asia and North America, and (4) the separation of Africa and South America. Black: Spinosauridae; red: Spinosaurinae; yellow: Baryonychinae. Dashed lines represent dubious Jurassic specimens and question marks indicate taxonomic uncertainty within Spinosauridae.

### 4.2. Dispersal potential in spinosaurids

Evidence suggests that spinosaurids consumed fish, but were not necessarily obligate piscivores, as indicated by the presence of juvenile Iguanodontid material within the gut contents of *Baryonyx* [80], and teeth referable to *Irritator* embedded within a pterosaur vertebra [81]. Association of fish remains with spinosaurid teeth [17], as well as isotopic evidence [82] and supraorbital salt glands in spinosaurines [83], suggest spinosaurids spent a significant amount of their time in aquatic environments. Moreover, morphological features of the skull, including the retracted external nares [13], ‘pelican-like’ spreading of the lower jaws in *Irritator* [84], and specialized neurovascular openings on the snout-tip [85], suggest at least some specialization towards aquatic prey capture [86]. Spinosaurids may have foraged in aquatic habitats more than other medium-to-large theropods within the same ecosystem, such as carcharodontosaurids and abelisaurids, therefore reducing inter-clade competition through dietary niche partitioning and differences in habitat usage [1,82,87–89]. Here, we report that spinosaurids have a close affinity to aquatic environments with relatively high MATs and relatively minimal seasonal temperature shifts, which may have constrained or influenced their dispersal potential (figure 3, tables 1 and 3). Although the cause for this apparent thermal preference remains to be tested, perhaps warmer climates provided favourable conditions for the aquatic ecosystems and prey which spinosaurids relied on.

There is debate among researchers on the ecology of the genus *Spinosaurus*, as to whether or not it was a subaqueous hunter [15,19], or a wading predator comparable to a heron [10,20,22]. Biomechanical work, evidence from CT data [20,21,24] and spinosaurids recovered from inland riparian deposits [23,51] support the wading predator hypothesis, yet some characteristics of *S. aegyptiacus*, such as a paddle-like tail and a pachyostotic skeleton, have been argued in favour of a subaqueous foraging ecology [5,16,19]. Recent biophysical models have found that transoceanic dispersal in Late Cretaceous hadrosaurs and titanosaur sauropods was possible for distances up to approximately 560 km, which was previously considered unlikely for the apparently terrestrially bound titanosaurs [90]. The supposed aquatic adaptations of spinosaurids may imply they had the potential to undergo transoceanic dispersals [5,16,19], however, biomechanicalmodels [20,21] suggest that spinosaurid swimming capabilities were unremarkable and therefore may have been insufficient in facilitating oceanic dispersal. Despite the disputed potential for transoceanic dispersal in spinosaurids, other barriers likely constrained their biogeography, such as their thermophysiology.

Across amniotes, the physiological and behavioural adaptations that stem from an organism's ability to control the loss and gain of heat influence their suitable ecospace occupation [91,92]. In modern active thermoregulators, the temperature of the environment independently requires maintenance of metabolic energy requirements, thereby influencing activities [92]. Elevated metabolic rates were likely ancestral to theropods owing to a highly efficient pulmonary anatomy [91,93], small erythrocytes [94], insulating integumentary structures [95] and an increased accumulation of S- and N-crosslinks, generating metabolically advanced lipoxidation end products [96]. This would contribute to year-round activity at high latitudes and elevations [97,98]. However, the absence of quantitative data surrounding spinosaurid growth rates, ancestral body size, along with the lack of consensus on the subaqueous nature of spinosaurids, even excluding *Spinosaurus* [15,18], limits our ability to accurately reconstruct spinosaurid thermophysiology. Furthermore, to facilitate the long periods of submerged aquatic locomotion necessary for transoceanic dispersal, spinosaurids would have required a novel suite of physiological characteristics, such as hyper-specialized vascular adaptations, to redirect blood flow from the periphery and to accommodate changes in pressure depending on whether the animal was actively swimming, diving or managing heat loss [99]. Owing to a lack of soft-tissue preservation and inadequate osteological correlates needed to reconstruct vascularization, a lack of specialized mechanisms for myoglobin oxygen storage or a lack of anatomical adaptations for achieving dynamic insulation in existing spinosaurid specimens, our ability to infer spinosaurid ecology is further obscured. Body mass and ecospace occupation (aquatic versus terrestrial environments) dramatically impact physiological temperature regulation, and new and more complete specimens may elucidate spinosaurid thermophysiology.

### 4.3. Biogeographic evolution

#### 4.3.1. Europe

Our analysis posits a European origin for Spinosauridae, alongside closely related megalosauroid clades, such as Megalosaurinae and Afroventatorinae (figure 2). Europe boasts some of the oldest undisputed spinosaur material, including an isolated tooth (BEXHM 1995.485) from the Valanginian Wadhurst Clay formation in South East England ([100]; figure 4), as well as several spinosaurids from the Wealden Group on the Isle of Wight [100] and multiple localities across the Iberian Peninsula [32]. Milner [74] suggested that dispersal between Europe and Africa may have involved Iberia, though the direction of dispersal remained unclear [101]. The potential for Iberia to facilitate a dispersal route has been supported by numerous spinosaurid fossils from this landmass [102], and our results indicate that spinosaurid dispersal likely occurred ‘out-of-Europe’. The exact route by which spinosaurids left Europe is unknown, although it may be possible that the clade crossed from Iberia, as suggested by Milner [74]. Alternatively, if spinosaurids were not adept at transoceanic dispersal [20,21], it is possible they migrated more manageably through island chains along the Apulian route ([103]; figures 1 and 2). Barker *et al.* [28] suggested dispersal to Asia and Gondwana occurred in the Early Cretaceous and two Europe–Africa dispersal events for spinosaurids, which our analyses also support (figure 2). Despite Europe being the cradle of spinosaurid diversification, the European spinosaur record disappears by the Aptian (figure 4). Although the reason for this is currently unclear, it may simply be a result of sampling failure, absence of suitable habitat, resources or preservational biases.

#### 4.3.2. North America

The absence of spinosaurid remains from North America is noteworthy, given the unambiguous presence of other megalosauroids on the continent [47,104]. While previous claims have been made that one specimen (BYU 2020) may be referable to Spinosauridae [33], other studies have tentatively referred this specimen to the megalosaurid *Torvosaurus* [47]. If the absence of spinosaurids from North America is not a case of taphonomic bias, it remains unclear whether geographic, climatic or other potential barriers inhibited their dispersal from Europe. The North Atlantic may have formed a substantial barrier to dispersal between North America and Europe during the Barremian [105]. However, more recent geologic interpretations indicate a possible land bridge may have formed between Europe and North America through Greenland and Fennoscandia via the Barents shelf during the late Hauterivian–early Barremian [105,106]. This land bridge complicates explanation for the absence of spinosaurids, although it may be possible that the cooler Greenland paleoclimate may have been unsuitable for the clade (figure 3; [70,107]) and prevented European individuals from dispersing into North America across this route.

#### 4.3.3. Africa and South America

Our results support the hypothesis that Gondwanan spinosaurids were derived from European populations. A land bridge via the Apulian route (a carbonate platform in the eastern Mediterranean region between northern Africa and southern Europe [108]) is hypothesized to have been present during the Berriasian–Barremian [106,109] and may have facilitated the movement of spinosaurids into Gondwana. Our results indicate that at least two waves of spinosaurid dispersal from Africa into South America likely occurred, based on the arrival of *Irritator* and *Oxalaia* in Brazil (figure 2; [110–112]), prior to the full separation of the two continents in the latest Albian (figure 2; [23]).

For South American megalosauroids, although a single possible Early Cretaceous occurrence is known from Argentina, the paucity of the material (a single tooth; [113]) warrants caution regarding as to its implications for the biogeographical dispersal of spinosaurids and megalosauroids in general. A possible constraint for spinosaur dispersal across Gondwana during the Early Cretaceous was the Central Gondwanan Desert (CGD), which was present in both South America and Africa [107,114]. However, the CGD likely waned and became progressively easier to cross by the earliest Cretaceous [64], potentially allowing spinosaurids to navigate it. Support for this comes from the occurrence of spinosaurid teeth from Brazil's Feliz Deserto formation, which are attributable to both Spinosaurinae and Baryonychinae [115–117]. The Feliz Deserto material may imply a possible third spinosaurine dispersal and a cryptic migration of baryonychines to the continent in the Early Cretaceous. Alternatively, the Feliz Deserto Spinosaurinae material may constrain the arrival of one of the spinosaurine lineages to the Early Cretaceous, if the material is ancestral to *Irritator* or *Oxalaia* (figures 2 and 4). The Berriasian–Valanginian Feliz Deserto specimens potentially represent the earliest spinosaurid material (figure 4). Despite this, a European origin for spinosaurids still remains likely, as the family diverged from a European megalosauroid ancestor (figure 2). Sections of desert may have persisted into the Albian within southwest Laurasia (i.e. modern Central America) and northernmost South America [107]; which may have acted as a barrier, perhaps alongside the proto-Caribbean seaway (if transoceanic migration was not possible [20,21]), to spinosaurid dispersal from Gondwana to North America.

#### 4.3.4. Australia, Antarctica and Indo-Madagascar

Antarctica, India and Australia have not yielded definitive spinosaurid material [52], but Middle Jurassic material from India has been suggested to possibly belong to the clade [58]. Material recovered from the Aptian–Albian Eumeralla formation of Australia, once thought to belong to Spinosauridae, has been subsequently identified as a Megaraptoran (cf. *Australovenator*; [46,118]). The absence of spinosaurids from these landmasses lends evidence to the idea that the clade likely did not reach Gondwana until the earliest Cretaceous (figure 4), when eastern Gondwanan landmasses (Australia, Indo-Madagascar and Antarctica) are thought to have fully separated from Africa [119]. Yet, issues of limited exposure and/or sampling intensity of Early to ‘Middle’ Cretaceous terrestrial deposits across Antarctica, India and Madagascar mean a sampling bias for these landmasses cannot be ruled out [120–122]. Climate model and proxy data [70,107] of southern South America, Australia and Antarctica suggest these landmasses also likely fell outside of the apparent MAT range of most spinosaurids (approx. 26.1–33.1°C; table 3; figure 3), which perhaps inhibited their dispersal. This may suggest that the absence of spinosaurid fossils from southernmost South America is the result of a genuine climatic barrier. Fossil evidence indicates that other Early Cretaceous dinosaurian clades, such as allosauroids, may not have been as restricted and may have dispersed from South America to Australia via Antarctica [72]. The differential dispersal capabilities of other dinosaur clades between South America and Australia suggest a unique combination of factors limiting spinosaurid dispersal compared with other theropods, such as a potentially constrained MAT range (figure 3), which could otherwise venture towards cooler, more poleward latitudes.

#### 4.3.5. Asia

The presence of *Ichthyovenator* reveals a dispersal event into Asia from Europe by the Aptian (figure 2), which was likely facilitated through the formation of an ephemeral land bridge during the lowering of the Uralian Seaway (figure 1; [52]). Other Asian spinosaur fossils [52,123] may indicate that dispersal into Asia was more complicated, but more material is required for robust phylogenetic analyses. The oldest spinosaurids from Asia are very fragmentary in nature and their placement in phylogeny is uncertain, including: a tooth (Yuasa formation, Japan [124]), the potentially dubious *Siamosaurus* [54], the ‘Hin Lat Yao spinosaurid’ [125], ‘Phuwiang spinosaurid B’ [126] and other indeterminate spinosaurid teeth (figure 4; [52]); all from the Sao Khua formation of Thailand. The age of the Sao Khua formation is debated, with palaeobiological proxies (i.e. vertebrates, non-marine bivalves and palynology) suggesting a late Barremian or earliest Aptian age [34,55,127–129], whereas radiometric dating of detrital zircons suggests a late Valanginian (approx. 137–132 Ma) to Hauterivian age (approx. 132–126 Ma) [130]. Resolving the taxonomic affinity and the age of this material would help to constrain the occurrence and timing of the dispersal(s) to Asia and elucidate the diversity of Asian spinosaurids. This uncertainty has important biogeographical implications, as it potentially highlights Northeastern Thailand as an Asian early spinosaurid diversification hotspot. A late Valanginian age would also constrain the arrival of the ancestral Asian spinosaurid population from Europe to before the second Early Cretaceous Uralian seaway regression (figure 4).

Baryonychines are notably absent from the Asian spinosaurid record, with no material confidently recognized from the continent (figure 4). The potential drivers for the apparent absence of baryonychines are currently unresolved, but we speculate that this could be a result of sampling bias, possible misidentification of material or may reflect a genuine biological signal (including competitive exclusion by endemic spinosaurine populations or physiological limitations). Yet, ‘Phuwiang spinosaurid B’ has been recovered as both a baryonychine [23] and a spinosaurine [125] using the same phylogenetic matrix [20], emphasizing the taxonomic uncertainty of this specimen within Spinosauridae. Putative baryonychine material (41HIII-00012; [131]) was recovered from the Cenomanian-aged Mangchuan formation of China, which, if accurately referred, would be one of the youngest members of the clade, extending the Laurasian spinosaur record [131] and supporting at least two spinosaurid dispersals to Asia [14]. Colder climates surrounding the Bering land bridge [70,107] may have prevented spinosaurid dispersal from Asia into North America, unlike the pattern seen in tyrannosauroid theropods that crossed between the late Aptian and late Albian [106,132,133].

#### 4.3.6. Late Cretaceous spinosaurids

Following their confirmed 50-million-year history, Spinosauridae eventually went extinct by, or near, the Cenomanian-Turonian boundary [9,134]. Potential post-Cenomanian spinosaurids have been recovered from Brazil [135], Patagonia [136], China [137] and Peru [138], however their referral to Spinosauridae is disputed and likely represent other sauropsid taxa [9,69,77,139–141]. The latest uncontested spinosaurids are *Spinosaurus* and *Oxalaia* from the Cenomanian of Africa and South America, respectively. While the cause of spinosaurid extinction is uncertain, it likely occurred during the Cenomanian–Turonian interval, which witnessed global sea-level transgression and oceanic anoxic events [142], as well as the hottest global temperatures in the last 200 million years [143]. The extinction of other dinosaurian clades, such as rebbachisaurids and carcharodontosaurids, which dominated ecosystems since the beginning of the Cretaceous [144,145], also occurred during this interval, followed by the diversification of other Late Cretaceous theropods [79]. In light of our analysis of possible habitat preferences in spinosaurids, these global climatic changes likely contributed to the extinction of the clade.

#### 4.3.7. Considering potential Jurassic spinosaurid specimens

Geologically earlier records of putative spinosaurids have been reported from the Middle–Late Jurassic (figure 4; [53,56–58]), although this material is sparse and their taxonomic affinities often ambiguous owing to either very close morphological similarities with crocodylomorphs and other theropod clades and/or misinterpretations of the anatomy of these specimens [9]. As some of these earlier specimens may potentially have significant implications for spinosaurid biogeographic history [69], such as an earlier arrival into Gondwana than suggested by our analyses (figure 4), we find these specimens worthy of further discussion. While alternative taxonomic referrals are increasingly favoured for these specimens, with recent morphological analyses suggesting these probably belong to Crocodylomorpha, Ceratosauria and Megalosauridae [76,77,146], the megalosaurid–spinosaurid divergence likely occurred in the Middle Jurassic [23], raising the potential for Jurassic-aged spinosaurid fossils to be discovered in geographically significant regions. Currently, the oldest definitive spinosaurid remains are Berriasian–Valanginian in age (as discussed above). Despite this, the spinosaurid ghost lineage suggests that even older spinosaurids remain to be discovered [23].

Although the spinosaurid affinities of these specimens are disputed, Jurassic spinosaurs may have existed and given rise to Cretaceous spinosaurid lineages. Here, we outline possible biogeographic scenarios raised by the hypothesis that these Jurassic specimens indeed represent Jurassic spinosaurids. It should be noted that these scenarios are, at present, hypothetical and somewhat speculative as there are currently no definitive Jurassic spinosaurids known. Further, these scenarios have no bearing on what has already been discussed for Cretaceous spinosaurid biogeography, as our primary results provide a conservative set of interpretations, owing to the exclusion of dubious taxa, such as those listed above. Descriptions of new species or more complete specimens of known taxa have the potential to change the results of these analyses. Here, we offer a hypothesis as to the spatio-temporal history of the earliest spinosaurids and the origin of the European lineages, which would give rise to the iconic *Baryonyx* and *Spinosaurus*. This hypothesis could be tested by the discovery of definitive Jurassic spinosaurid body fossils and testing their phylogenetic position within the clade.

Potential spinosaurids from the Bathonian of India and Niger [56,58], the Kimmeridgian of Tanzania (*Ostafrikasaurus*; [53]) and the Tithonian of France [59] suggest that spinosaurids may have originated in Gondwana [53,57] and dispersed northwards into Europe by the latest Jurassic. It may be the case that the earliest spinosaurids were not specifically adapted for a (semi-)piscivorous diet relative to the megalosaurian ancestor, and this more generalized early spinosaurid ecology may have allowed putative Jurassic specimens, such as *Ostafrikasaurus*, to stray into drier areas, such as the CGD, thereby facilitating wider dispersal during their early evolution. Ecological similarities between megalosaurids and early spinosaurids may have prevented their coexistence, perhaps forcing spinosaurid ancestors to disperse and diversify out of Europe, which may be supported by the aforementioned spinosaurid teeth from the Sao Khua formation of Thailand, which has recently been debated to be as old as the Valanginian [130], and from the South American Feliz Deserto formation, which may be as old as the Berriasian [115–117]. Following the likely extinction of megalosaurids at the end of the Jurassic, spinosaurids may have dispersed back toward European biotas, where the origin and diversification of definitive European Spinosauridae occurred.

Alternatively, basal spinosaurids did not leave Europe but were instead rare components of Jurassic ecosystems as a result of competition with incumbent megalosaurids. This may explain the apparent scarcity of European Jurassic spinosaurid material, which currently consists of a single dubious tooth [59], despite being a reasonably well-sampled continent. Indeed, it has been suggested that some megalosaurids may have also explored piscivory, with potential evidence including an association of fish remains with *Dubreuillosaurus* [147], the skull morphology of *Eustreptospondylus* [148], and trackways suggestive of coastal foraging [149], which may support the hypothesis of competition between the two clades. Strong similarities between megalosaurids and early spinosaurids may also explain the latter's absence from Jurassic North America, as their dispersal across the proto-Atlantic was impeded by the presence of megalosaurids in Late Jurassic Iberia [4]. A connection between Iberia and North America is thought to have been present during the late Kimmeridgian and early Tithonian (approx. 154–151 Ma; [105]), which would have facilitated the arrival of megalosaurids to the USA [47] alongside other Iberian species, including turiasaurian sauropods [150,151] and an interchange of other genera between the two continents (such as *Allosaurus*, *Stegosaurus* and *Diplodocus*; [106,152–155]).

Conversely, the absence of definitive spinosaurid material from the Jurassic may indicate that Spinosauridae (in its current understanding) descended from Jurassic megalosaurids, suggesting that Megalosauridae may be a paraphyletic lineage of megalosauroids, leading to spinosaurids. Rauhut & Pol [49] indeed recovered Megalosauroidea as paraphyletic; their phylogenetic analysis recovered spinosaurids as earlier-diverging than megalosaurids, potentially disputing this hypothesis. However, the absence of Jurassic spinosaurids from the fossil record is most likely an artefact of spatio-temporal sampling failure, suggesting that we are still yet to sample the earliest representatives of this enigmatic clade of theropods and that more intensive sampling efforts in undersampled regions, such as the Jurassic of Asia and Africa, may be key in unravelling the evolutionary history of Spinosauridae.

#### 4.3.8. Study limitations

The consistent recovery of the DIVALIKE or DIVALIKE+J models as best describing megalosauroid biogeography indicates that the uncertainty in spinosaurid relationships does not majorly influence interpretations of overall spinosaurid biogeography, as the uncertainty largely relates to the degree of long-distance or ‘jump’ dispersal between models. Future phylogenetically or biogeographically important specimens could refine our models and the implications of dispersal. It should be noted that differences between palaeoclimate modelling and proxy data have been recognized when applied to the same spatio-temporal context [156–160]. As such, it is possible that environmental reconstructions from proxy data may indicate different inhabited climatic regimes for spinosaurids. It should be noted that the biological signal of these proposed climate preferences may be confounded by sampling artefacts, restricting our spinosaurid sample to particular climatic/environmental regimes. Future discoveries may alter the apparent broad-scale climatic patterns we observe (figure 3).

Taxonomically unstable and uncertain spinosaurid material currently cannot be included in the BioGeoBEARS analysis, as the software package requires a fully resolved phylogeny to function. Instead, we attempt to account for the different evolutionary scenarios of more stable taxa by running multiple phylogenetic trees. Even if it were possible to include more taxonomically unstable and uncertain specimens, these would likely only affect the number of potential dispersals and are unlikely to have a substantial impact on the results, as this additional material is recovered from continents where spinosaurids are already reported, and not in regions where they are yet to be discovered.

## 5. Conclusion

We have demonstrated that the evolutionary history of spinosaurid theropods was largely influenced by vicariance of widespread ancestral populations, as well as constrained dispersal following the removal/emplacement of climatic and physical barriers. Our results indicate that spinosaurid dispersal between landmasses may have been limited by mechanisms involving environmental factors, such as a preference for warmer, semi-aquatic, habitats and geographical obstacles, including marine barriers. Based on currently available data, Europe remains the most likely origin for spinosaurids and served as the cradle for the evolutionary radiation of these enigmatic theropods (figure 5).

**Figure 5.**
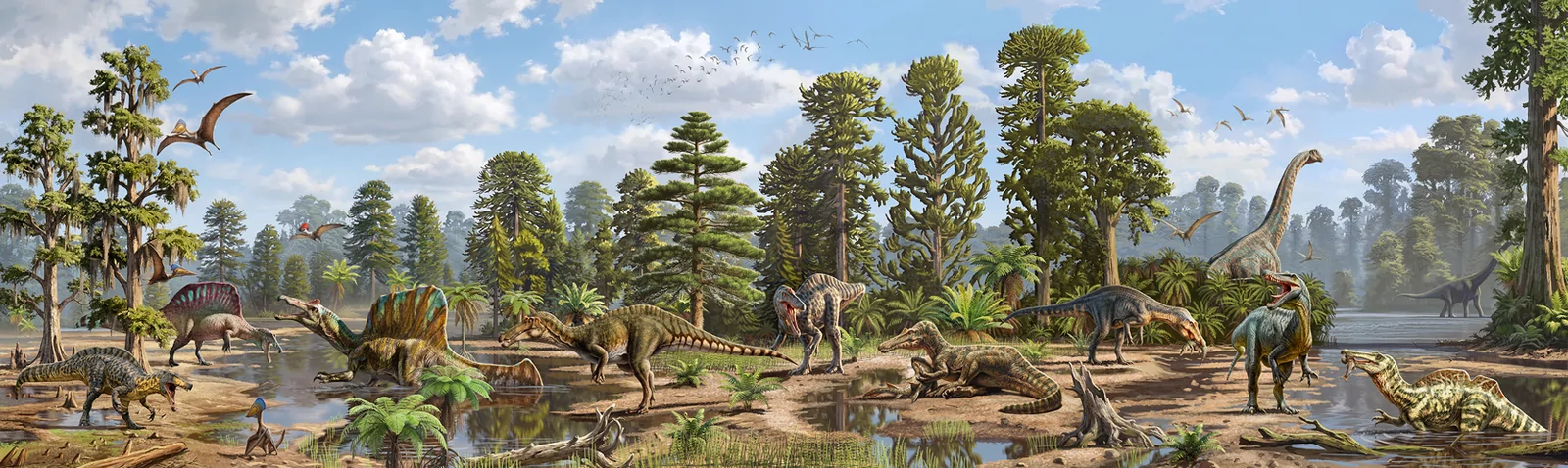
Relatives from across the ponds. An illustration of Cretaceous spinosaurid diversity, each in their respective palaeoenvironments. From left to right: South America (*Irritator*, *Oxalaia*); Africa (*Spinosaurus*, *Suchomimus*); Europe (*Vallibonavenatrix*, *Riparovenator*, *Ceratosuchops*, *Baryonyx*); and Asia (*Ichthyovenator*). Artwork by Pedro Salas and Sergey Krasovskiy.

## Supplementary Material



## Data Availability

All data are included in the supplementary material [161].
