# Supplementary material for: Sailing to Gondwana: multiple waves of spinosaurid dispersal to the southern continents
Source: R Soc Open Sci. 2026 Oct 28;13(10):260994. doi: 10.1098/rsos.260994 (PMC13714370; doi:10.1098/rsos.260994)
Supplement: Supplementary file 1 [file rsos.260994.zip › rsos.260994/rsos260994_si_001.zip!/Supplementary Info/Supplementary Information.docx]

**Sailing to Gondwana: Multiple waves of spinosaurid dispersal to the southern continents – Supplementary Information**

Tree 1: Baryonychinae [As per Barker et al., 2021], Spinosaurinae [Arden et al., 2019], Riojavenatrix [As Baryonychine per Rauhut et al. 2025], Iberospinus [basal spinosaurid per Mateus & Estraviz-López, 2022], Camarillasaurus [As basal spinosaurid per Sereno et al., 2022], White rock spinosaur [As basal spinosaurine per Barker et al. 2022], Vallibonavenatrix as an early spinosaurine [As per Rauhut et al. 2025]

Tree 2: As tree 1, but with Iberospinus as the earliest spinosaurid [As per Mateus & Estraviz-López, 2022].

Tree 3: As tree 1, but with Iberospinus and white rock spinosaurid as earliest spinosaurids [As per Barker et al. 2022].

Tree 4: As tree 1, but with Iberospinus and white rock spinosaurid as earliest spinosaurids; Riojavenatrix as earliest Spinosaurinae [As per Rahut & Pol, 2021].

Tree 5: As tree 1 but with Iberospinus and white rock spinosaurid as earliest spinosaurids; Riojavenatrix as earliest spinosaurinae; and Camarillasaurus as sister to Spinosaurus [As per Rauhut et al. 2025].

Tree 6: As tree 1, but with Iberospinus and white rock spinosaurid as earliest spinosaurids; Riojavenatrix as earliest spinosaurinae; Camarillasaurus as sister to Spinosaurus and Sigilmassasaurus as sister to Irritator [Resolved by *multi2di*].

Figure S1

Figure S1: The BioGEOBEARS tree based on tree topologies 2 and dispersal matrix 1.

Figure S2

Figure S2: The BioGEOBEARS tree based on tree topologies 3 and dispersal matrix 1.

Figure S3

Figure S3: The BioGEOBEARS tree based on tree topologies 4 and dispersal matrix 1.

Figure S4

Figure S4: The BioGEOBEARS tree based on tree topologies 5 and dispersal matrix 1.

Figure S5

Figure S5: The BioGEOBEARS tree based on tree topologies 6 and dispersal matrix 1.

Figure S6

Figure S6: The BioGEOBEARS tree based on tree topologies 1 and dispersal matrix 2.

Figure S7

Figure S7: The BioGEOBEARS tree based on tree topologies 2 and dispersal matrix 2.

Figure S8

Figure S8: The BioGEOBEARS tree based on tree topologies 3 and dispersal matrix 2.

Figure S9

Figure S9: The BioGEOBEARS tree based on tree topologies 4 and dispersal matrix 2.

Figure S10

Figure S10: The BioGEOBEARS tree based on tree topologies 5 and dispersal matrix 2.

Figure S11

Figure S11: The BioGEOBEARS tree based on tree topologies 6 and dispersal matrix 2.

**Table S1**

| **Model** | **LnL** | **Ln likelihood ratio** | | **AICc analysis** | | |
| --- | --- | --- | --- | --- | --- | --- |
|  |  | D-statistic | *p*-value | AICc | AICc_wt_ | Ratio |
| DEC | -56.14 | - | - | 116.7 | 0.13 | - |
| DEC+J | -54.86 | 2.57 | 0.11 | 116.6 | 0.14 | 1.076923077 |
| **DIVALIKE** | **-55.03** | **-** | **-** | **114.5** | **0.41** | **-** |
| DIVALIKE+J | -54.03 | 2 | 0.16 | 115 | 0.31 | 0.7804878049 |
| BAYAREALIKE | -70.76 | - | - | 146 | 6.0e-08 | - |
| BAYAREALIKE+J | -65.3 | 10.92 | 0.0010 | 137.5 | 4.1e-06 | 6.83E+01 |

Table S1: Summary statistics from BioGeoBEARS analysis using tree topology 2 and dispersal matrix 1.

Table S2

| **Model** | **LnL** | **Ln likelihood ratio** | | **AICc analysis** | | |
| --- | --- | --- | --- | --- | --- | --- |
|  |  | D-statistic | *p*-value | AICc | AICc_wt_ | Ratio |
| DEC | -55.17 | - | - | 114.8 | 0.072 | - |
| DEC+J | -53.98 | 2.38 | 0.12 | 114.9 | 0.068 | 0.9444444444 |
| **DIVALIKE** | **-53.37** | **-** | **-** | **111.2** | **0.43** | **-** |
| DIVALIKE+J | -52.14 | 2.45 | 0.12 | 111.2 | 0.43 | 1 |
| BAYAREALIKE | -70.17 | - | - | 144.8 | 2.20E-08 | - |
| BAYAREALIKE+J | -65 | 10.34 | 0.0013 | 136.9 | 1.10E-06 | 5.00E+01 |

Summary statistics from BioGeoBEARS analysis using tree topology 3 and dispersal matrix 1.

Table S3

| **Model** | **LnL** | **Ln likelihood ratio** | | **AICc analysis** | | |
| --- | --- | --- | --- | --- | --- | --- |
|  |  | D-statistic | *p*-value | AICc | AICc_wt_ | Ratio |
| DEC | -62.71 | - | - | 129.9 | 0.0084 | - |
| DEC+J | -58.81 | 7.8 | 0.0052 | 124.5 | 0.12 | 14.28571429 |
| DIVALIKE | -59.94 | - | - | 124.3 | 0.13 | **-** |
| **DIVALIKE+J** | **-57** | **5.89** | **0.015** | **120.9** | **0.74** | **5.692307692** |
| BAYAREALIKE | -74.79 | - | - | 154 | 4.80E-08 | - |
| BAYAREALIKE+J | -71 | 7.57 | 0.0059 | 148.9 | 6.10E-07 | 1.27E+01 |

Summary statistics from BioGeoBEARS analysis using tree topology 4 and dispersal matrix 1.

Table S4

| **Model** | **LnL** | **Ln likelihood ratio** | | **AICc analysis** | | |
| --- | --- | --- | --- | --- | --- | --- |
|  |  | D-statistic | *p*-value | AICc | AICc_wt_ | Ratio |
| DEC | -63.62 | - | - | 131.7 | 0.0082 | - |
| DEC+J | -59.3 | 8.65 | 0.0033 | 125.5 | 0.18 | 21.95121951 |
| DIVALIKE | -62.22 | - | - | 128.9 | 0.033 | - |
| **DIVALIKE+J** | **-57.83** | **8.78** | **0.003** | **122.6** | **0.78** | 23.63636364 |
| BAYAREALIKE | -74.76 | - | - | 154 | 1.20E-07 | - |
| BAYAREALIKE+J | -69.68 | 10.15 | 0.0014 | 146.3 | 5.60E-06 | 4.67E+01 |

Summary statistics from BioGeoBEARS analysis using tree topology 5 and dispersal matrix 1.

Table S5

| **Model** | **LnL** | **Ln likelihood ratio** | | **AICc analysis** | | |
| --- | --- | --- | --- | --- | --- | --- |
|  |  | D-statistic | *p*-value | AICc | AICc_wt_ | Ratio |
| DEC | -62.71 | - | - | 129.9 | 0.0084 | - |
| DEC+J | -58.81 | 7.8 | 0.0052 | 124.5 | 0.12 | 14.28571429 |
| DIVALIKE | -59.94 | - | - | 124.3 | 0.13 | - |
| **DIVALIKE+J** | **-57** | **5.89** | **0.015** | **120.9** | **0.74** | 5.692307692 |
| BAYAREALIKE | -74.79 | - | - | 154 | 4.80E-08 | - |
| BAYAREALIKE+J | -71 | 7.57 | 0.0059 | 148.9 | 6.10E-07 | 1.27E+01 |

Summary statistics from BioGeoBEARS analysis using tree topology 6 and dispersal matrix 1.

Table S6

| **Model** | **LnL** | **Ln likelihood ratio** | | **AICc analysis** | | |
| --- | --- | --- | --- | --- | --- | --- |
|  |  | D-statistic | *p*-value | AICc | AICc_wt_ | Ratio |
| DEC | -55.58 | - | - | 115.6 | 0.12 | - |
| DEC+J | -54.36 | 2.43 | 0.12 | 115.7 | 0.12 | 1 |
| **DIVALIKE** | **-54.32** | **-** | **-** | **113.1** | **0.43** | **-** |
| DIVALIKE+J | -53.33 | 1.98 | 0.16 | 113.6 | 0.33 | 0.7674418605 |
| BAYAREALIKE | -70.09 | - | - | 144.6 | 6.10E-08 | - |
| BAYAREALIKE+J | -61.92 | 16.34 | 5.30E-05 | 130.8 | 6.20E-05 | 1.02E+03 |

Summary statistics from BioGeoBEARS analysis using tree topology 1 and dispersal matrix 2.

Table S7

| **Model** | **LnL** | **Ln likelihood ratio** | | **AICc analysis** | | |
| --- | --- | --- | --- | --- | --- | --- |
|  |  | D-statistic | *p*-value | AICc | AICc_wt_ | Ratio |
| DEC | -55.58 | - | - | 115.6 | 0.12 | - |
| DEC+J | -54.36 | 2.43 | 0.12 | 115.7 | 0.12 | 1 |
| **DIVALIKE** | **-54.32** | **-** | **-** | **113.1** | **0.43** | **-** |
| DIVALIKE+J | -53.33 | 1.98 | 0.16 | 113.6 | 0.33 | 0.7674418605 |
| BAYAREALIKE | -70.09 | - | - | 144.6 | 6.10E-08 | - |
| BAYAREALIKE+J | -61.92 | 16.34 | 5.30E-05 | 130.8 | 6.20E-05 | 1.02E+03 |

Summary statistics from BioGeoBEARS analysis using tree topology 2 and dispersal matrix 2.

Table S8

| **Model** | **LnL** | **Ln likelihood ratio** | | **AICc analysis** | | |
| --- | --- | --- | --- | --- | --- | --- |
|  |  | D-statistic | *p*-value | AICc | AICc_wt_ | Ratio |
| DEC | -54.93 | - | - | 114.3 | 0.082 | - |
| DEC+J | -53.9 | 2.07 | 0.15 | 114.7 | 0.066 | 0.8048780488 |
| **DIVALIKE** | **-53.2** | **-** | **-** | **110.8** | **0.46** | **-** |
| DIVALIKE+J | -52.13 | 2.15 | 0.14 | 111.2 | 0.39 | 0.847826087 |
| BAYAREALIKE | -69.54 | - | - | 143.5 | 3.70E-08 | - |
| BAYAREALIKE+J | -61.97 | 15.15 | 9.90E-05 | 130.9 | 2.10E-05 | 5.68E+02 |

Summary statistics from BioGeoBEARS analysis using tree topology 3 and dispersal matrix 2.

Table S9

| **Model** | **LnL** | **Ln likelihood ratio** | | **AICc analysis** | | |
| --- | --- | --- | --- | --- | --- | --- |
|  |  | D-statistic | *p*-value | AICc | AICc_wt_ | Ratio |
| DEC | -62.35 | - | - | 129.1 | 0.008 | - |
| DEC+J | -58.29 | 8.12 | 0.0044 | 123.5 | 0.13 | 16.25 |
| DIVALIKE | -59.92 | - | - | 124.3 | 0.091 | - |
| **DIVALIKE+J** | **-56.55** | **6.74** | **0.0095** | **120** | **0.77** | 8.461538462 |
| BAYAREALIKE | -74.12 | - | - | 152.7 | 6.20E-08 | - |
| BAYAREALIKE+J | -68.69 | 10.85 | 0.001 | 144.3 | 4.10E-06 | 6.61E+01 |

Summary statistics from BioGeoBEARS analysis using tree topology 4 and dispersal matrix 2.

Table S10

| **Model** | **LnL** | **Ln likelihood ratio** | | **AICc analysis** | | |
| --- | --- | --- | --- | --- | --- | --- |
|  |  | D-statistic | *p*-value | AICc | AICc_wt_ | Ratio |
| DEC | -63.49 | - | - | 131.4 | 0.057 | - |
| DEC+J | -61.25 | 4.48 | 0.034 | 129.4 | 0.15 | 2.631578947 |
| DIVALIKE | -62.36 | - | - | 129.2 | 0.18 | - |
| **DIVALIKE+J** | **-59.87** | **4.98** | **0.026** | **126.7** | **0.61** | 3.388888889 |
| BAYAREALIKE | -74.32 | - | - | 153.1 | 1.10E-06 | - |
| BAYAREALIKE+J | -70.34 | 7.96 | 0.0048 | 147.6 | 1.70E-05 | 1.55E+01 |

Summary statistics from BioGeoBEARS analysis using tree topology 5 and dispersal matrix 2.

Table S11

| **Model** | **LnL** | **Ln likelihood ratio** | | **AICc analysis** | | |
| --- | --- | --- | --- | --- | --- | --- |
|  |  | D-statistic | *p*-value | AICc | AICc_wt_ | Ratio |
| DEC | -62.35 | - | - | 129.1 | 0.008 | - |
| DEC+J | -58.29 | 8.12 | 0.0044 | 123.5 | 0.13 | 16.25 |
| DIVALIKE | -59.92 | - | - | 124.3 | 0.091 | - |
| **DIVALIKE+J** | **-56.55** | **6.74** | **0.0095** | **120** | **0.77** | 8.461538462 |
| BAYAREALIKE | -74.12 | - | - | 152.7 | 6.20E-08 | - |
| BAYAREALIKE+J | -68.69 | 10.85 | 0.001 | 144.3 | 4.10E-06 | 6.61E+01 |

Summary statistics from BioGeoBEARS analysis using tree topology 6 and dispersal matrix 2.

Table S12:

| Clade Comparison | MAT (p-value) | ST (p-value) | MAP (p-value) | SP (p-value) |
| --- | --- | --- | --- | --- |
| Spinosauridae ~ Carcharodontosauria | **0.0001142** | 0.198 | 0.9255 | 0.265 |
| Spinosauridae ~ Abelisauridae | **0.02716** | 0.1508 | 0.3203 | 0.2765 |
| Spinosauridae ~ Metriacanthosauridae | 0.07189 | 0.4876 | 0.6102 | 0.5431 |
| Spinosauridae ~ Megaraptora | **0.009371** | **0.0001677** | 0.1328 | 0.2584 |
| Spinosauridae ~ Noasauridae | 0.6544 | **0.02725** | 0.09519 | 0.1514 |
| Spinosauridae ~ Tyrannosauroidea | **2.582e-10** | **4.471e-08** | 0.05213 | 0.3928 |
| Spinosauridae ~ Dromaeosauridae | **< 2.2e-16** | **< 2.2e-16** | 0.0677 | 0.5016 |
| Spinosauridae ~ Troodontidae | **3.411e-12** | **6.723e-11** | 0.6572 | 0.1977 |
| Spinosauridae ~ Avialae | **< 2.2e-16** | **< 2.2e-16** | 0.9656 | **0.01139** |
| Spinosauridae ~ Ornithomimosauria | **1.261e-12** | **8.847e-12** | 0.8472 | 0.0634 |
| Spinosauridae ~ Therizinosauria | **3.393e-09** | **1.779e-09** | 0.5888 | **0.04845** |

Tables S12: Non-parametric Mann-Whitney U tests between clades and their climate ranges. Significant results (*p* < 0.05) are emboldened. The data used for this table comes from Chiarenza et al. (2022).

References:

Arden, T.M., Klein, C.G., Zouhri, S. and Longrich, N.R., 2019. Aquatic adaptation in the skull of carnivorous dinosaurs (Theropoda: Spinosauridae) and the evolution of aquatic habits in spinosaurids. Cretaceous Research, 93, pp.275-284.

Barker, C.T., Hone, D.W., Naish, D., Cau, A., Lockwood, J.A., Foster, B., Clarkin, C.E., Schneider, P. and Gostling, N.J., 2021. New spinosaurids from the Wessex Formation (Early Cretaceous, UK) and the European origins of Spinosauridae. Scientific Reports, 11(1), p.19340.

Barker, C.T., Lockwood, J.A., Naish, D., Brown, S., Hart, A., Tulloch, E. and Gostling, N.J., 2022. A European giant: a large spinosaurid (Dinosauria: Theropoda) from the Vectis Formation (Wealden Group, Early Cretaceous), UK. PeerJ, 10, p.e13543.

Chiarenza AA, Mannion PD, Farnsworth A, Carrano MT, Varela S. 2022. Climatic constraints on the biogeographic history of Mesozoic dinosaurs. Curr Biol. 32, 570-585.

Mateus, O. and Estraviz-López, D., 2022. A new theropod dinosaur from the early cretaceous (Barremian) of Cabo Espichel, Portugal: Implications for spinosaurid evolution. PLoS One, 17(2), p.e0262614.

Rauhut, O.W. and Pol, D., 2021. New theropod remains from the late Jurassic Cañadón Calcáreo formation of Chubut, Argentina. Journal of South American Earth Sciences, 111, p.103434.

Rauhut, O.W., Canudo, J.I. and Castanera, D., 2025. Revision of the theropod dinosaur Camarillasaurus cirugedae from the Early Cretaceous (Barremian) of Teruel province, Spain (No. ART-2025-145831).

Sereno, P.C., Myhrvold, N., Henderson, D.M., Fish, F.E., Vidal, D., Baumgart, S.L., Keillor, T.M., Formoso, K.K. and Conroy, L.L., 2022. Spinosaurus is not an aquatic dinosaur. Elife, 11, p.e80092.
